# OMICs Approaches in Diarrhetic Shellfish Toxins Research

**DOI:** 10.3390/toxins12080493

**Published:** 2020-07-31

**Authors:** Alexandre Campos, Marisa Freitas, André M. de Almeida, José Carlos Martins, Dany Domínguez-Pérez, Hugo Osório, Vitor Vasconcelos, Pedro Reis Costa

**Affiliations:** 1 CIIMAR-Interdisciplinary Centre of Marine and Environmental Research, University of Porto, Terminal de Cruzeiros do Porto de Leixões, Av. General Norton de Matos, s/n, 4450–208 Porto, Portugal; maf@ess.ipp.pt (M.F.); jmartins@ciimar.up.pt (J.C.M.); dany.perez@ciimar.up.pt (D.D.-P.); vmvascon@fc.up.pt (V.V.); 2 ESS-P.Porto, School of Health, Polytechnic Institute of Porto. Rua Dr. António Bernardino de Almeida, 400, 4200-072 Porto, Portugal; 3 LEAF-Instituto Superior de Agronomia, Universidade de Lisboa, 1349-017 Lisboa, Portugal; aalmeida@isa.ulisboa.pt; 4 i3S–Instituto de Investigação e Inovação em Saúde, Universidade do Porto, 4200-135 Porto, Portugal; hosorio@i3s.up.pt; 5 Ipatimup—Instituto de Patologia e Imunologia Molecular da Universidade do Porto, 4200-135 Porto, Portugal; 6 Faculdade de Medicina, Universidade do Porto, 4200-319 Porto, Portugal; 7 Biology Department, Faculty of Sciences, University of Porto, Rua do Campo Alegre, s/n, 4169-007 Porto, Portugal; 8 IPMA—Instituto Português do Mar da Atmosfera, Rua Alfredo Magalhães Ramalho, 6, 1495-006 Lisbon, Portugal; prcosta@ipma.pt

**Keywords:** diarrhetic shellfish toxins, aquatic contamination, mechanisms of toxicity, risk assessment, transcriptomics, proteomics, metabolomics

## Abstract

Diarrhetic shellfish toxins (DSTs) are among the most prevalent marine toxins in Europe’s and in other temperate coastal regions. These toxins are produced by several dinoflagellate species; however, the contamination of the marine trophic chain is often attributed to species of the genus *Dinophysis*. This group of toxins, constituted by okadaic acid (OA) and analogous molecules (dinophysistoxins, DTXs), are highly harmful to humans, causing severe poisoning symptoms caused by the ingestion of contaminated seafood. Knowledge on the mode of action and toxicology of OA and the chemical characterization and accumulation of DSTs in seafood species (bivalves, gastropods and crustaceans) has significantly contributed to understand the impacts of these toxins in humans. Considerable information is however missing, particularly at the molecular and metabolic levels involving toxin uptake, distribution, compartmentalization and biotransformation and the interaction of DSTs with aquatic organisms. Recent contributions to the knowledge of DSTs arise from transcriptomics and proteomics research. Indeed, OMICs constitute a research field dedicated to the systematic analysis on the organisms’ metabolisms. The methodologies used in OMICs are also highly effective to identify critical metabolic pathways affecting the physiology of the organisms. In this review, we analyze the main contributions provided so far by OMICs to DSTs research and discuss the prospects of OMICs with regard to the DSTs toxicology and the significance of these toxins to public health, food safety and aquaculture.

## 1. Introduction

Diarrhetic shellfish toxins (DSTs) are frequently the most abundant and recurrent toxins derived from harmful algal blooms (HABs) in Europe (North Atlantic) [1], as well as other temperate regions of the world such as Asia or South America. In Portugal, for example, outbreaks of harmful algae occur every year, between April and October [2] having as one of the main consequences the banning of shellfish harvest and trade. DSTs are produced by several species of dinoflagellates belonging to the genera *Dinophysis* and *Prorocentrum* [3], being however the species of the genus *Dynophysis* the main source of DSTs in the marine trophic chain [3]. DSTs are a broad group of molecules that include okadaic acid (OA) and analogous molecules named dinophysistoxins (DTX-1, -2 and -3) [3]. Moreover, these toxins have lipophilic properties and tend to accumulate in the adipose tissues, being therefore difficult to metabolize and eliminate [4]. The main mechanism of action of DSTs is the inhibition of serine/threonine protein phosphatases 1 and 2A (PP1 and PP2A), causing deregulation of several intracellular processes [5]. The main route of transmission of DSTs to humans is the consumption of contaminated seafood, and among seafood, shellfish are known to accumulate the highest toxin levels. Indeed, DSTs pose a significant concern to food safety as they affect human health, causing acute food poisoning (e.g., diarrhea, nausea, vomiting and abdominal pain) [6,7]. To overcome this public health issue, several measures have been implemented in many countries including the regular monitoring of shellfish production and harvesting areas, the establishment of regulatory limits for DSTs in seafood and temporary bans on shellfish harvesting during HABs occurrence.

Marine biotoxins and DSTs in particular have received great attention from the scientific community, due to the concerns on the impact of these toxins in human health and the environment. Research has mainly focused on understanding the biology and growth of DSTs producing phytoplankton species, their dynamics in the environment and mechanisms of adaptation to environmental stress factors [8,9,10,11]. The knowledge obtained has been useful for instance to improve monitoring and forecast toxic microalgae outbreaks and also to predict new patterns of geographical distribution of DSTs in the context of climate change. The focus has been also the chemical characterization of the several toxin analogues (OA and DTXs), including the esterified forms accumulated by shellfish [12,13], and the development of more sensitive and portable instruments applied in environmental monitoring (e.g., development of biosensors) [14,15]. The toxicology of DSTs has been also investigated. Most relevant research topics include DSTs mechanisms of action, molecular and cellular targets and toxicokinectics. Other relevant research topics are the transfer of DSTs in the food chain and DSTs bioaccessibility and bioavailability [16,17]. Most of the studies carried out in these topics are contributing to improve the prevention and/or mitigation of the adverse effects of DSTs in human health, environment and aquaculture and fisheries.

A noteworthy advance was achieved in understanding DSTs toxicology in humans. Indeed, it is now possible to describe with some detail the mode of action of OA and to explain the cytotoxic and neurotoxic effects of this group of toxins [18,19], as well as some associated pathologies such as cancer [20,21]. The knowledge obtained in this research field of human toxicology is, in part, due to OMICs research, which has allowed to resolve some OA toxicity mechanisms, which were not possible to obtain using other research methodologies. Unfortunately, other research areas lack similar functional and molecular understanding. These research areas are dedicated to the toxicology of aquatic vertebrates and invertebrates, trophic transfer, bioaccumulation and biomagnification of DSTs in the marine food chain and DSTs biotransformation. For example, the enzymes and genes involved in the biotransformation of DSTs in bivalves (mainly acylation reactions) were not yet identified, despite the importance of such knowledge for the assessment of oral toxicity of seafood and for understanding the kinetics of DSTs depuration.

**Figure 1:**
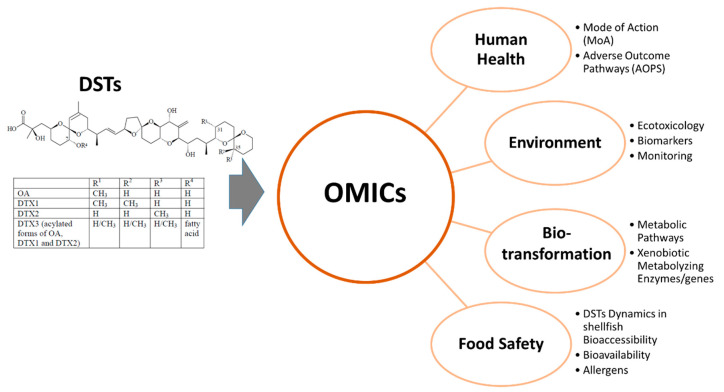
Research areas covered by OMICs contributing to risk assessment of DSTs. General structure of diarrhetic shellfish toxins (DSTs) and respective legend table were adapted from Larsen et al. (2007) [22].

This review is dedicated to the emergent field of OMICs in the context of marine biotoxins research. It is particularly focused in the DSTs group and aims to provide an overview of the research carried out to date involving OMICs approaches and the outcomes of such research related with risk assessment, as detailed in Figure 1. Furthermore, we aim to discuss future avenues of OMICs research in the fields of marine biotoxins and human health, ecotoxicology and food safety.

## 2. OMICs Overview

OMICs comprehends a growing number of disciplines in the field of life sciences. Different OMICs may be found in the literature. They include for instance transcriptomics, proteomics or metabolomics. Each different OMIC refers to a certain “OME’”. For instance, proteomics concerns the study of the proteome, which can be defined as all the proteins that exist (or are detectable) in a given organelle or cellular compartment, cell, tissue, organ, fluid, organism or population. In an analogous way, the metabolome concerns to all metabolites found in a given organelle or cellular compartment, cell, tissue, organ, fluid, organism or population. OMICs require high-throughput technologies and each different OMIC uses a specific set of analytical technologies. For instance, transcriptomics was first based on the use of arrays [23] and later evolved into the use of next generation sequencing (NGS) of DNA and RNA [24,25]. Similarly, proteomics that was first based on two-dimensional gel electrophoresis with protein identification using mass spectrometry (MS) later evolved into gel-free techniques able to quantitate large numbers of proteins using MS. For instance, high-throughput LC-MS/MS, combined with isotope-coded affinity tags (ICAT) labelling and peptide enrichment, enabled to describe proteomic alterations related with phosphorylation and OA inhibition of PP2A and PP1 [26]. Finally, metabolomics uses two major technologies: NMR (Nuclear Magnetic Resonance) and MS. It is noteworthy however that numerous other OMICs are also available such as peptidomics, lipidomics or phosphoproteomics. For a review on the different OMICs and their specific approaches and methodologies, readers are directed to our recent publication on the subject [27].

Either combined or individually, OMICs are a formidable approach to analyze biological systems, particularly when contrasting two opposing situations that an organism is subjected to. For instance, considering this very simple example: a mussel species in a control environment and subjected to an algae toxin. The use of proteomics would allow researchers to determine the proteins that would have higher accumulation in response to the algal toxin exposure and consequently infer the effect of the toxin on the different biochemical pathways, ultimately contributing to a deeper knowledge of the bio-toxic effects of the toxin. If the proteomics, investigation is combined with other OMICs, and such combination allows a very integrative and highly complementary approach, providing an in-depth view on the biological effects of that toxin on the mussel species. The same rationale can enable the discovery of biomarkers of exposure to the toxin, which in sentinel species such as mussels could be used to obtain information about the occurrence of DSTs outbreak [28].

In such a context, either alone or combined, OMICs have been extensively used in a plethora of different subjects within life sciences. These include human health [29], animal sciences [30] and aquatic and environmental sciences [31,32]. Numerous reviews may be found in the subject and certain OMICs, namely proteomics, has been pointed out as being of particular relevance for animal, veterinary and aquatic science [33,34,35].

Nevertheless, OMICs can hardly be seen as the silver bullet that answers all biological questions. Indeed, there are numerous aspects that limit their use. Ultimately, the success or failure of an OMICs approach is heavily dependent on both technical and biological limitations. One of such limitations is the availability of high-quality databases. Indeed, and generally speaking, well-studied or model organisms have a wide range of characterized transcripts, proteins or metabolites in research databases whereas poorly studied organisms do not. Such a low level of representation in the databases can be a severe problem as it limits the amount of identified proteins, metabolites or transcripts. This problem is evident in farm animals [36] and aquatic species [37], where those with fully sequenced genomes such as the marine mussel (*Mytilus galloprovincialis*) [38] or oyster (*Magallana gigas,* old name *Crassostrea gigas*) [39] have better characterized proteome or transcriptome databases than other species like cockles (*Ceratosderma edule*), clams (*Ruditapes decussatus*) or the marine gastropod *Lobatus gigas* [31,40,41]. This problem is not as relevant in metabolomics as the metabolites are chemically very similar regardless of the studied organism. Another relevant limitation is the fact that the same transcripts, proteins and metabolites tend to accumulate in response to different types of stressors, creating a déjà vu impression in OMICs literature [42,43]. The problem was particularly relevant in gel-based proteomics where the differentially accumulated proteins had to be physically noted in the two gel, and these tended to be the same (or at least with a high homology) between species. A third important problem has to do with the robustness of the statistical power analysis. This aspect is frequently overlooked, particularly in complex organisms where it is very difficult to establish homogenous experimental groups due to the high variability between individuals [44]. As an example, an experimental group of eight mice of a certain inbred strain is much more homogenous than a group of eight mussels collected from the shore. Despite these limitations, OMICs approaches are gaining importance and are becoming a very important tool to address life-sciences research. Indeed, and as time progresses since the onset of technologies over the last 20 years, databases are becoming increasingly robust, and the reagent costs that were initially very expensive and specific are becoming more affordable and widespread. Similarly, the instrumentation use costs are also becoming more affordable as generalist OMICs platforms are established across the world. These platforms provide different state of the art OMICs services at very reasonable prices, without the need for individual research groups to purchase costly instruments or hire specialized staff that would always have a sub-optimal use. This is particularly relevant in countries and institutions where access to these technologies are not so common [45]. In the subsequent sections, we will overview the usefulness of OMICs in aquatic sciences research, taking particular attention to the case of DSTs and the impact of these toxins in the human health and environment.

## 3. Human Toxicology of DSTs

The inhibition of PP1 and PP2A is generally accepted as the key initiating event of OA and DTXs toxicity. The inhibition of PP1 and PP2A by OA was first reported by Bialojan and Takai in 1988 [46], and since then, studies provided growing evidences of OA inhibition of PP1/2A. OA in particular has been thoroughly studied in the scope of DSTs toxicology. In addition, OA has been a very useful molecule in cell biology research, used as a specific inhibitor of PP1 and PP2A, to understand protein phosphorylation, cell signaling cascades mediated by phosphorylation and the role of PPs in the cell [47,48].

Following the discovery of PP inhibition, the binding of OA to PP1 and PP2A was investigated and characterized [49,50]. Moreover, toxicological studies revealed OA and DSTs both cytotoxic and genotoxic effects. More recently, OMICs approaches have been contributing to the elucidation of the molecular events associated to OA toxicity and to understand the pathologies associated to this group of toxins that include for instance cancer [21,51]. One of the most reported molecular event associated to PP inhibition is the hyper-phosphorylation of cytoskeleton proteins. These alterations have been linked to cell morphological rearrangement and other adverse outcomes such as apoptosis. Related to this, a proteomics study targeting specialized cell membrane areas called lipid rafts was developed to gain insights on the effects of OA related to the cytoskeleton. The authors reported significant alterations both at the phosphorylation level and the abundance of several proteins from such lipid rafts [52]. Among those were microtubule-associated Ser/Thr-protein kinase 4 (STK4) or talin-2, plectin-1 (PLEC1), α-spectrin (SPTA) and MARCKS-related protein, demonstrating that OA can effectively impair the interaction of cytoskeleton with cell membrane and affect cell-to-cell adhesion and communication processes [52]. Moreover, this process can be triggered by the interaction of OA with PP2A, since this enzyme seemed to be associated with lipid rafts functions [52]. OMICs can also help in revealing cell-type related toxicity mechanisms. For instance, villin (VIL), an actin-binding protein present in microvilli filaments, was pointed out as one of the proteins implicated in the loss of microvilli architecture in small intestine cells affected in mouse orally exposed to OA. This protein was inhibited by OA, along with several other proteins involved in macromolecular metabolism, cytoskeleton reorganization, signal transduction, molecular chaperoning and oxidative stress [53].

The interactome of PPs can also provide information about the mode of action of OA and DSTs. A proteomics study carried out by Yadav et al. (2017) [48], combining unique quantitative affinity and mass spectrometry methods, enabled an in-depth characterization of the dynamics of PPs complex assemblies. With this quantitative affinity approach, 66 PPs (54 catalytic and 12 regulatory) belonging to three major families, phosphoprotein phosphatases (PPPs), protein phosphatases Mg^2+^ (PPMs) and protein tyrosine phosphatases (PTPs), were investigated leading to the identification of 631 new protein interactions involving members of the three PP families.

The study revealed that OA can change PP1 and PP2A substrate-affinity and their interactions with other proteins, by altering the phosphorylation patterns of PP1 and PP2A. The study also revealed that OA did not affect the main PP1 and PP2A core modules; nevertheless, interactions of the core modules with other proteins changed significantly, affecting the overall PPs interactome structure [48].

With regard to PP2 interactome, OA led to the dissociation from the interactome of several proteins, whose functions are related with striatin complex, snRNA binding and pre-mRNA splicing. The striatin complex plays a relevant role in the cell by acting as scaffolding unit in multiple large signaling complexes, including PP2A [54]. These results indicate that by interacting with PP2A, OA may indeed affect a variety of cellular processes. Moreover, the dissociation of the molecular chaperone T-complex protein 1 (TCP1) with several PP2A regulatory subunits with OA was also observed [48]. Since some of these regulatory subunits are TCP1 substrates, these results suggest that OA may affect the folding and stabilization of these regulatory subunits and consequently PP2A activity in the cell. OA impaired the association of protein phosphatase methylesterase 1 (PPME1) to PP2A scaffolding subunit 2AAA, adding evidences that PPME1 is also a target of OA and competes with the binding site of PP2A catalytic subunit. OA also altered the association of the complexes coiled-coil domain-containing protein 6 (CCDC6)-PP4C with PP2A. These modifications have shown to be important to DNA damage response and loss of CCDC6 could lead to genome instability and cancer development [55].

With regard to PP1 complex, the study also revealed that OA affects PP1 interactions with unconventional RPB5 interactor 1 (URI) complex. URI is a chaperone complex, involved in the regulation of transcription [56]. It is also thought to be involved in the regulation of S6 kinase-mediated signaling and mitochondrial apoptosis mechanisms [57]. In addition, PP5 interactions with molecular chaperones such as heat-shock proteins (HSP90A, HSP90B), HSP90 co-chaperones (TEBP and CDC37) were enhanced up to 1.7-fold and new interactions with STIP1 and H90B4 chaperones occurring in the presence of OA [48], suggesting that OA affects PP5 mediated chaperonin functions in the cells. Interactions of PP5 with protein argonaute (AGO1, AGO3) were also increased, and new interactions with pre-mRNA-processing-splicing factor 8 (PRP8), U5 small nuclear ribonucleoprotein (U520) and polynucleotide 5′-hydroxyl-kinase (NOL9) proteins suggest that OA interfere with PP5 functions in RNA-processing and small-RNA-guided gene silencing. Although not considered a target of OA, the toxin indeed affected PPM1G complex composition. This PP formed a large complex with different core histones and glutamate-rich WD repeat-containing protein 1 (GRWD1), suggesting that OA may alter some of PPM1G key functions on histone modification and DNA-damage response [48].

Proteomics also revealed alterations in phosphorylation of key regulatory proteins such as HSP27, a molecular chaperone known to mediate different biological processes including phosphorylation through protein kinase binding [58] and protein kinases MAPK, ERK1/2, GSK3B and CAMK2 involved in signaling cascades closely related with the development of neurotoxicity and neurodegeneration [47].

The effects of OA, DTX-1 and another type of marine toxin named azaspiracid-1 (AZA-1) were compared in a microarray study. The study revealed effects in mRNA expression very similar between OA and DTX-1. Gene expression revealed alterations in genes involved in hypoxia induced factor (HIF) processes, forkhead box protein O (FOXO) and sterol regulatory element-binding protein (SREBP) signaling, endoplasmic reticulum (ER) stress and unfolded protein response (UPR), DNA methylation, telomere maintenance, cell cycle and detoxification [59].

Gene expression studies revealed induction of genes of the canonical Wnt/β-catenin-signaling pathway, at non-cytotoxic OA concentrations in HepaRG cells. The results shade some light on OA carcinogenic mechanisms. β-catenin (CTNNB1), a key member of the pathway, was affected at the transcription and post-transcription levels and cellular location [60].

Toxicity of OA in liver was linked with the down-regulation of the transcription factor *NRF2* and some of its target genes with antioxidant functions, superoxide dismutase (*SOD1*), catalase (*CAT*) and heme oxygenase 1 (*HMOX1*) and detoxification and ribosyldihydronicotinamide dehydrogenase (*NQO*). The down-regulation of these genes can lead to the accumulation of reactive oxygen species (ROS) and liver toxicity [61]. In addition, the link between cancer initiation/progression and OA exposure was investigated by gene expression. The study revealed early alterations in gene expression involving genes from the anaphase-promoting complex (APC), the oncogene pituitary tumor-transforming gene 1 (*PTTG1*) and clusterin (*CLU*). APC genes are known to mediate metaphase to anaphase transition, whereas *PTTG1* has been associated to mitotic disruptions and chromosome separation inhibition [62]. *CLU* gene encodes a chaperone involved in several molecular processes including proteasome degradation, regulation of cell proliferation and apoptosis [62].

The toxicological information of DSTs is crucial to understand the impact of these compounds in human health. Much has been studied with regard to OA, but relatively few information has been collected about the toxicology of DTX-1, -2 and -3. Most of the studies revolve around the determination of DTXs acute toxicity (LD50) by intraperitoneal (i.p.) injection [63], but studies on chronic and sub-toxic exposure are still missing. Chemical differences may confer to DTXs bioactivities different from OA. We also verify the absence of toxicological studies on the combined action of OA and DTXs, despite the frequent involvement of OA and several DTXs in food poisoning. We anticipate that OMICs will have a role to play in the differentiation of the bioactivities of the different DSTs and in the assessment of their toxic potential. Highly sensitive transcriptomics and proteomics methodologies may help to characterize chronic and sub-toxic effects of DSTs.

## 4. Ecotoxicological Effects of DSTs

The impact of DSTs on aquatic organisms has been poorly studied so far. Nevertheless, marine vertebrates and invertebrates have been found to be strongly affected by DST-producing microalgae and their toxins [64]. Among the main aquatic groups of organisms, bivalve mollusks are those that received more attention regarding toxicity of OA-group toxins. The importance of the bivalve shellfish industry worldwide and the many problems that the development of this sector faces are well known. A significant issue is related with the occurrence of HABs and resulting contamination of shellfish with marine biotoxins. Research interest in the mitigation of phycotoxins from raw live shellfish has continuously increased in recent years due to concerns over economic losses, food safety and human health. Thus, the biological effects generated by DSTs on commercial bivalves have been addressed in a large number of studies, particularly when compared to other aquatic organisms. In this context, OMICs (transcriptomics and proteomics) have been applied, especially in recent years, in the description of the cellular processes involved in the bivalve’s response to OA and OA-producing microalgae toxicity.

Filter-feeding bivalves are the main vectors of OA and its analogues, showing differences between species in the capacity of DSTs uptake and elimination. These lipophilic toxins are mainly accumulated in the digestive gland [65,66] and several studies showed that DSTs undergo a series of molecular transformations during the digestive process (for review see [67]). Shell-valve closure response, pseudo-feces production and clearance rate reduction are among the strategies described for oysters (*M. gigas*) and mussels (*Perna perna, M. edulis*) to deal with increasing densities of OA-producing dinoflagellates (*P. lima, D. acuminata*) [68,69,70,71]. Nevertheless, histopathological alterations along with immunotoxicity, cytotoxicity and genotoxicity effects have been shown to be induced by DST-producing microalgae or their purified toxins. Despite all the biological impacts, no records of mortality have been associated to bivalves naturally exposed to DST-dinoflagellates blooms. Many studies suggest that these mollusks have developed cellular mechanisms of protection to overcome the harmful effects of phycotoxins. The involvement of the first line defense antioxidants (such as SOD, CAT and GPx) [72], phase-I and -II drug metabolizing enzymes cytochrome P450 (CYP450) and glutathione s-transferase (GST) [73,74] and ATP-binding cassette (ABC) transporters (e.g., P-glycoprotein) [75,76] have been related to *P. lima* and associated toxins resistance in bivalves.

OMICs approaches, mostly toxicogenomics, have been applied in bivalves experimentally exposed to purified OA and to the OA-producing dinoflagellate *P. lima* to describe the molecular pathways behind the toxicity of DSTs. One of the first studies used a mussel cDNA microarray to evaluate gene expression changes in the digestive gland of *M. galloprovincialis* fed for five weeks with OA-contaminated food (6.5 µg of OA every third day) [77]. Interestingly, the results evidenced the presence of two distinct phases in the response to OA. An early response (3-days post-treatment) was characterized by a high number of up-regulated genes, suggesting an activation of putative defense mechanisms and/or physiological adjustments. In contrast, the latter time points (21- and 35-days post-treatment) were marked by an escalating transcriptional repression suggesting the disruption of several cellular processes caused by the accumulation of the toxin [77]. Taken together, the most represented up- regulated transcripts categories could all be linked to OA- induced cytoskeleton destabilization (e.g., actin, precollagen-P), apoptosis (e.g., factors inducers of apoptosis, regulators of the apoptosis/prohibitin system, homologues of the C-myc proto-oncogene, ubiquitin C-variant, Int-6) and stress/immune-responses (e.g., heat shock proteins, particularly *HSP90*) [77]. It should be noted however that in this study, 51% of the total overexpressed transcript sequences showed no significant similarities in the public nucleotide and protein databases [77]. More recently, the bay scallop *Argopecten irradians* was also exposed to purified OA (500 nM) and the transcriptomic responses of gills tissue evaluated after 48h exposure using deep-sequencing technology (Illumina HiSeq 4000) [78]. Gene ontology classification and enrichment analysis of the differentially expressed genes (DEGs) revealed four ontologies with particularly high functional enrichment, namely “cellular process” (cellular component), “metabolic process” (biological process), “immune system process” (biological process), and “catalytic process” (molecular function). The OA-responsive genes included the NADPH oxidase 3 (*NOX-3*) transcripts, a relevant source of ROS generation and *HSP70* transcripts, involved in the protection of cells from ROS [79,80]. Besides these stress-related genes, DGEs were mostly related to a series of detoxification and immune processes in the response to OA [78]. Immune-related genes such as Toll-like receptor, cyclic AMP-responsive element-binding protein and acid phosphatase were all up-regulated. Likewise, detoxification-related genes including CYP450 (*CYP3A4* and *CYP3A80*) and ABC transporters (*ABCB10*, *ABCC5* and *ABCC1*) were up-regulated, in contrast with Phase II GSTs (*GST1*, *GST2*, *GST-A*, *GST-Theta-1*, *GST-Omega* and *GST-Kappa*). It was suggested that the downregulation of GSTs is partially compensated by the upregulation of Cu/Zn SOD, since both enzymes use the same substrate [78].

To better understand the molecular impact of DSTs, several other studies have been exploring bivalve’s response to the epiphytic dinoflagellate *P. lima*. Thus, Huang et al. (2015) [28] applying a 2D-electrophoresis approach found that most of the differentially expressed proteins in the gills of *Perna viridis* after 6h exposure to this toxic dinoflagellate (1 × 10^6^ cell L^−1^) were involved in metabolism. Among these, proteins such as ubiquitin-conjugating enzyme E2 and SINA-like protein were up-regulated suggesting that *P. lima* induced ubiquitination/proteasome activity. On the other hand, proteins such as aldolase (ALD), malate dehydrogenase (MDH) and isocitrate dehydrogenase (IDH) were down-regulated, suggesting that *P. lima* might induce the disruption of energy metabolism in mussels [28]. *P. lima* exposure significantly changed expression of other proteins, which are potentially involved in other physiological processes in mussels, including cytoskeleton assembling, signal transduction and detoxification. Within the latter category, cytochrome P450 and ABC transporters were up-regulated, which was consistent with OA-exposure-induced gene expression of both proteins in *A. irradians* gills [28,78]. The early response to DSTs was also studied in mussels exposed to low microalga cell densities, simulating an early stage DSTs bloom. Suarez-Ulloa et al. (2015) [81] showed that exposure to 200 cells L^−1^ of *P. lima* for 24 h was enough to induce gene expression modifications in both digestive gland and gills of the mussel *M. galloprovincialis* using NGS and custom-made microarray technologies combined. No major functional differences were found when comparing the profiles for both tissues, although absolute differences in magnitude are evident between digestive gland and gill, being more dramatic in the former [81]. Transcriptomic analyses revealed an increase in proteasomal activity, molecular transport, cell cycle regulation, energy production and immune activity in mussels. Interestingly, a number of transcripts associated to OA toxicity like the specific targets PP1 and PP2A and multi-xenobiotic resistance proteins failed to show significant changes in expression [81]. In a similar study, Prego-Faraldo et al. (2018) [82] assessed the early response (48 h) of the same mussel to *P. lima* (1 × 10^5^ cell L^−1^) using a RNA-Seq analysis showing, in this turn, a higher number of DGEs in gills compared to digestive gland. These included transcripts involved in defense, immunity and metabolism, and since many of them have been described as making part of a common cellular stress response of bivalves to other stimuli [83], it is suggested that mussel’s resistance mechanism to DSTs is to some extent unspecific [82]. Furthermore, results also seem to support a key role of the extracellular exosome and respiratory chain in both mussel tissues in the response to DSTs. The substantial down-regulation of genes related to the metabolic processes in digestive gland and apoptotic processes in gills, which in the latter case is not consistent with previous results obtained for OA [77], is also proposed as a primary harmful effect of DSTs on these tissues [82]. More recently, a *de novo* transcriptome analysis was performed in the digestive glands of *P. viridis* after exposure to *P. lima* (2 × 10^6^ cells L^−1^) for different time periods in order to gain more information about the defense mechanisms against DSTs [84]. Different changes in expression occurred at 6 h and 96 h after exposure to *P. lima* along with no significant differences in mussels’ OA content in digestive glands between both time points. The *de novo* transcriptome analysis combined with qPCR results suggests a progressive induction response during *P. lima* whole period of exposure through the activation of NRF2/ARE-dependent signaling pathway and downstream genes including phase II detoxification enzymes and ABC transporters (e.g., *GST-sigma 3*, *ABCB10*, *ABCG-like*) [84]. Furthermore, the changes in expression of *CYP3A-like* and *CYP2J2-like* suggests that cytochrome P450 enzymes might be involved in DSTs metabolism. The response to *P. lima* was also characterized by the down-regulation of immune system related genes such as *mgC1q83* and *mgC1q29* (after 6 h) and macrophage migration inhibitory factor (MIF) (after 96 h). In the latter stage (96 h) the up-regulation of cytoskeletal tubulin (*TUBA1C* and *TUBB1*) and anti-apoptotic genes (*IAP*—inhibitor of apoptosis proteins) might indicate compensatory mechanisms against DSTs [84].

Regarding other marine organisms, little is known about the effects and metabolism of DSTs. It is thus not surprising the almost non-existence of studies involving OMICs research regarding this matter for other organisms than bivalves. A few exceptions are related with the study of OA-induced effects in fish. Concerning these aquatic vertebrates, DSTs (dissolved and crude extracts) are known to induce severe damage in embryonic and larval stages of several fish species, from sub-lethal (e.g., inhibition of both protein and alkaline phosphatases) to lethal effects [85,86]. The OA-group toxins are also known to negatively impact the behavior of some studied juvenile and adult fish species, causing oxidative stress, histological alterations and even death [87,88,89]. Stress-related behaviors include (1) hyperactivities (jumps, fast left-right turns, surface swims), (2) poor feeding reflexes and abstinence from feeding, (3) lower metabolic fitness (oxygen consumption) and (4) reduction of swimming performances (these latter under increased stimulus) [87,88,89]. Liver and gills are especially affected by OA exposure in fish, with species like seabream *Sparus aurata* showing vascular dilation and hepatocellular membrane disintegration in the former and hypertrophy in secondary lamellae and necrotic aspect in the primary lamellae in the latter [87,88]. To provide clues on the potential molecular mechanisms of OA in fish, Zhang et al. (2014) [90] evaluated the transcriptomic responses induced by acute exposure to this lipophilic toxin in zebrafish liver tissues using a microarray approach. Most of the DEGs with significant fold change (beyond the range from −2 to 2) are related with signaling pathways (e.g., MAPK-related genes) and could result from the inhibition of the PP1 and PP2A by OA [90]. Other key genes differentially expressed after OA exposure included detoxification-related genes such as GSTs (*microsomal GST 1*) and ABC transporters (*ABCA3* and *ABCA5*), which were downregulated. Furthermore, genes involved in immune response such as *DNAJB9* (a member of *HSP40*) and *HSP70* showed a strong up-regulation in zebrafish liver, in contrast with *HSP90*. Interestingly, no significant differential expression of genes encoding PP1/2A was observed in zebrafish liver upon exposure to OA [90].

On a final note, though still sparsely used, it is clear by the above-mentioned studies the importance of OMICs application to the assessment of cellular responses behind DSTs toxicity on marine organisms. Mostly used on bivalves, OMICs evidence certain vulnerabilities of these mollusks to OA-producing microalga blooms but, simultaneously, have also been use to generate new insights on how these organisms counteract DSTs toxicity through crucial defense molecular mechanisms. A still common problem in OMICs research, which has been also evidenced by these studies, is the lack of genetic background information in databases which negatively affects genes/proteins identification and the consequent interpretation of responses as a whole.

## 5. Food Safety

**Figure 2:**
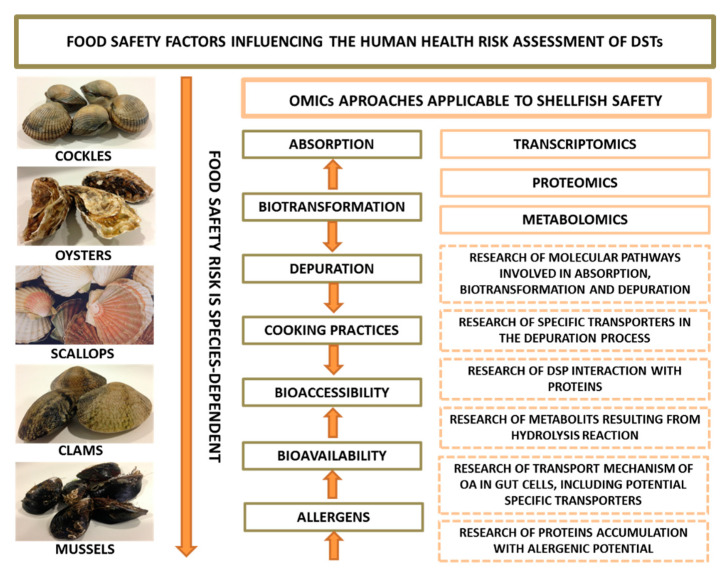
Food safety factors influencing the human health risk assessment of DSTs and OMICs research applications.

The consumption of seafood contaminated with OA and its analogues DTX-1, -2 and -3 [91] represents a food safety threat and, consequently, a public health concern due to their predicted increased occurrence as a consequence of climate change. Edible shellfish, crabs, snails and fish can accumulate toxins of the OA group [92,93]; however, filter-feeding shellfish (mussels, oysters, scallops, clams, cockles and others) seem to be the main vectors for human consumers [92,93,94]. Nevertheless, health risks of human acute or chronic exposure may depend on the species consumed, once the toxicity profile of contaminated shellfish seems to vary among species. According to Reguera et al. (2014) [3] shellfish contamination with DSTs results from a complex balance between (1) food selection, (2) absorption, (3) biotransformation, (4) elimination and (5) allometric processes (Figure 2). Different shellfish species exposed to the same DSTs producing algae appear to reveal differences in toxin accumulation [3,67]. Filtration rate can partially explain the differences in shellfish DSTs accumulation, as it seems to be very species-specific (e.g., mussels > oysters) [67]. Moreover, the extent to which DSTs are accumulated in shellfish depends on the balance between absorption and elimination processes [3]. The absorption of DSTs involves cellular uptake mechanisms and can occur through two main processes: (1) directly from the dissolved phase and (2) from the filtered microalgae cells or particulate matter that contain the toxins [67]. After absorption, DSTs undergo anatomical compartmentalization in the organism and elimination (detoxification) at different rates, depending again on the bivalve species (Figure 2). The elimination of DSTs involves (1) the removal of the toxins contained in non-digested microalgae cells and food materials (referred to weakly bound toxins) through the production of feces and pseudo-feces and (2) biotransformation and cellular excretion. Biotransformation has shown to be necessary for the elimination of toxins that were assimilated in shellfish tissues (internalized in the cells) [67]. The biotransformation of DSTs is discussed in more detail in the next section of this review, but briefly, following assimilation by digestive gland cells, DSTs undergo biotransformation processes (e.g., esterification of OA and DTX-2; hydrolysis of DTX-3) that depend on the chemical structure of each toxin [3]. The main biotransformation process that occurs in toxins of OA group is the esterification with saturated and unsaturated fatty acids (FA), forming 7-O-acyl derivatives (DTX-3), as previously found in scallop *Patinopecten yessoensis* [67,95], which have different toxic potential than their parent toxins [96]. Furthermore, acylation seems to be an important requirement for detoxification of DSTs in shellfish, being the balance between the rates of acylation and elimination of the acyl-derivatives what determines the efficiency of the depuration process [3]. DSTs are mainly depurated in esterified form, as this chemical form facilitates the molecular elimination of the toxin from the interior of the cells. Therefore, a poor esterification leads to a lower depuration rate of DSTs [67]. Studies carried out with the mussel *M. galloprovincialis* have shown that DTX-2 esterifies at a lower percentage than OA, thus depurates slowly [97,98]. Moreover, toxin elimination at the cell level is thought to involve specific membrane transport mechanisms. Membrane transporters of the ATP-Binding Cassette (ABC) family can be involved in the elimination of DSTs (including the transformed forms) accumulated in shellfish tissues. Gene and protein expression studies demonstrated that the membrane transporter P-GP, is expressed in different organs of bivalves after its exposure to *P. lima* [76]. Indeed, the expression and activity of this protein showed to increase with the accumulation of OA in gill tissues, suggesting that it can also have a role in the elimination of DSTs [75]. In addition, Suárez-Ulloa et al. (2015) [81] found an overexpression of genes related to vesicle-mediated transport in the mussel digestive gland after exposure to OA, suggesting that vesicular transport could contribute to cellular elimination. Indeed, recently, the potential contribution of vesicular transport to depuration was also advocated by Blanco et al. (2018) [67].

In general, the studies referring the dynamics of DSTs in shellfish have demonstrated that depuration rates of these toxins vary according to the following order of magnitude: cockles > oysters > scallops > clams > mussels [3,67,97,98,99,100]. With regard to esterification and depuration mechanisms, OMICs could contribute to identify the main transforming enzymes and to understand the specificity and selectivity of these enzymes to OA and DTX-1, -2. Sequence differences in these enzymes, if observed, could also help to explain the differences in the efficiency found among shellfish species in DSTs being metabolized and eliminated.

Regarding allometric processes, although these do not exert an influence in toxin accumulation, they can alter the toxin concentration in bivalves, thereby changing its toxic potential. For instance, starvation and spawning during and/or after an episode of contamination leads to a decrease in biomass and, in turn, to an increase of toxin concentration in shellfish tissues [3]. The metabolic alterations underlying these processes are poorly described. Nonetheless, they are of interest to understand the factors influencing the absorption, biotransformation and elimination of DSTs in shellfish. Thus, the development of studies applying OMICs technologies should be privileged, being an asset to understand the molecular processes involved in the absorption (uptake), biotransformation and elimination of DSTs, as well as the mechanisms of regulation of such processes in shellfish (Figure 2).

An important research topic related with food safety regards the interaction of DSTs with other biomolecules, particularly from the matrix of contaminated organisms (e.g., proteins and lipids). Despite the little attention given to the topic, one can expect it to have strong implications for food safety as it can lead to misleading estimations of shellfish toxicity as well as in the bioaccessibility/bioavailability of these toxins. It seems particularly relevant to understand what amount of the total toxin accumulated in shellfish is bound specifically to proteins and if this amount associated to proteins is being considered in the estimation of shellfish toxicity. DSTs are known to have high affinity to PP1 and PP2A. The binding to PPs is recognized as the key event triggering the subsequent processes that lead to toxicity. OA does not establish covalent bounds with PPs [46,49]; nevertheless, not much is known about the nature and stability of these bounds. In addition to PPs, no other molecular targets were so far described. Nonetheless, and to the best of our knowledge, no specific studies concerning the interaction of DSTs with proteins have been carried out. Toxin binding to different types of proteins is possible as toxins are molecules with increased biological activity and able to interact with different types of biomolecules. In this respect, a proteomic study revealed for instance that azaspiracids (AZAs), another type of lipophilic toxins produced by marine dinoflagellates, could indeed be found associated to different proteins. The authors related this toxin-protein association with prolonged AZA retention in shellfish tissues and the unusual low rates of depuration [101]. The nature of toxin–protein bounds is not known, but crosslinks or other type of chemical and ionic interactions cannot be excluded. OMICs and in particular proteomics employ powerful methods that enable the analysis of protein modifications and the characterization of protein binding with other substances or chemical groups [102,103,104,105]. Similar approaches can be employed in this case for investigating the putative associations of DSTs with shellfish proteins. In the case of DSTs being largely associated to proteins, this can influence the toxins bioaccessibility and consequently its bioavailability, mainly due to proteolytic digestion, which could promote the release of DSTs from the food matrix, making them available for absorption by intestinal cells. Furthermore, since before ingestion food is normally cooked, particular attention must be given to food processing procedures (e.g., steaming and autoclaving/canning), as usually these procedures lead to significant chemical and biochemical alterations in the protein content (e.g., protein aggregation, precipitation, crosslink) and thereby may change toxin availability. Currently, the food safety assessment of shellfish products commercially available for human consumption is made by direct comparison between the legal limit, 160 µg of OA equivalent kg^−1^ of shellfish meat [106], and the toxin levels determined in raw food matrix. Moreover, toxin levels are calculated considering the toxicity equivalent factor (TEF) of OA, DTX-1 and -2 and fatty acid esters derivatives (DTX-3). However, previous studies have shown that the DSTs content in shellfish is modified after these food products are subjected to common cooking practices (steaming and autoclaving/canning). The majority of the studies on this subject reported increased levels of OA and DTX2 in mussels after steaming and autoclaving [16,66,107,108]. In addition, the cooking fluids revealed substantial quantities of OA and DTX2 [66,107]. Nevertheless, reduced toxicity was observed (by 15%) in gastropod tissues subjected to industrial canning treatment (steaming 1 min at 121 °C, followed by autoclaving 5 min at 121 °C) [109]. Likewise, the bioaccessibility of OA toxin and its analogues, particularly in cockles, was also significantly decreased after steaming [16]. In fact, recent studies have shown that the bioaccessibility of DSTs is species-dependent (mussels > clams > cockles), and it is permanently less than 100%, which means that the human exposure to these contaminants is lower than the concentration presented in raw food [16,110]. Such differences could be related to the water loss from shellfish meat (increasing the toxicity) or the loss of DSTs from shellfish fluids, which would be leaked into the cooking water/packing media of canned products (decreasing the toxicity of shellfish meat). Still in this context, the influence of the associations of these toxins with proteins from shellfish tissues and fluids cannot be discarded and could be studied by applying proteomics studies.

Moreover, differences between matrices regarding the content and type of FA (saturated, monounsaturated and polyunsaturated) may affect the hydrolysis reaction that converts fatty acid esters (DTX-3) into their parent toxins (e.g., OA), changing the shellfish toxicity, as it was found in the bioaccessible fraction of mussel samples [16,110]. Therefore, metabolomics approaches, associated with conventional analytical methods [111], can be applied to generate information about the toxic potential of shellfish depending on the FA content and the metabolites generated after hydrolysis due to human digestion.

The bioavailability of OA has also been studied using in vitro intestinal Caco-2 cell models, and at low concentrations of exposure, the permeability to OA has been shown to be limited, mainly due to a significant efflux of OA, suggesting that active transport mechanisms are involved in the absorption restriction [112]. As the effects at the cellular level are reflected on gene and protein expression, OMICs studies on this topic would be extremely useful to elucidate the transport mechanism of OA in human gut cells, including the discovery of potential specific transporters [112].

Finally, given that DSTs can produce significant alterations at the protein level in contaminated organisms, affecting in particular the phosphorylation patterns as a consequence of the inhibition of PP1 and PP2A, this could be of relevance in terms of food safety, as it can affect the content of allergenic proteins. Allergenic proteins pose particular food safety concerns in crustaceans (prawns/shrimps, lobster, crab, crayfish), and several proteins including tropomyosin and arginine kinase have been related to allergic reactions to seafood [113]. Tropomyosin, paramyosin and arginine kinase have also been reported as protein allergens in mollusks [114]. The link between DSTs and allergenic proteins was not yet investigated. However, a study with blue mussel *M. edulis* exposed to a mix of 8 alkylphenols revealed several proteins affected by these contaminants [115]. The proteins differently expressed by mussels were involved in the cellular structure, metabolism and defense, being tropomyosin one of the down-regulated proteins identified [115]. The authors associated this alteration with a probable response to oxidative stress. In fact, many studies correlated the alteration of cytoskeleton proteins in relation with the production of ROS. Given that DSTs can also cause oxidative stress in shellfish (as documented in Section 3 and Section 4), it is likely that cytoskeleton proteins like tropomyosin and paramyosin can also be altered in abundance and structure by DSTs via induction of ROS and subsequently alter the allergenic potential of the animal. Furthermore, the study of Prego-Faraldo et al., (2018) [82] on the transcriptome of *M. galloprovincialis* revealed responses to DSTs involving defense and immune mechanisms and alterations in energy metabolism and ATP synthesis proteins. Alterations in the levels of ATP may affect another allergenic protein, arginine kinase, that uses ATP to phosphorylate arginine, in the metabolism of arginine and proline [114].

In this regard OMICs methods are particularly useful to undertake systematic analysis of this group of proteins and therefore to assist in the evaluation of the allergenic potential of seafood. In Figure 2, we summarize the main research topics on food safety and human health related with shellfish contamination with DSTs, as well as the main contributions expected from OMICs.

## 6. Shellfish Metabolism/Biotransformation of DSTs

The involvement of xenobiotic detoxifying enzymes and antioxidant system has been investigated by a considerable number of studies [72,116]. Most of these studies highlight protective mechanisms and expression of biochemical activities that make shellfish tolerate blooms of OA-producing dinoflagellates. However, the process underlying shellfish transformation of OA and related DSTs is not completely understood.

The toxic dinoflagellates, i.e., *Dinophysis* spp. and *Prorocentrum* spp. produce, store and transform themselves a suite of OA related compounds. Inactive toxins precursors, namely sulfated diesters, have been identified in cultures of *Prorocentrum* dinoflagellates [117,118,119,120]. These compounds, named DTX-4 and DTX-5, have been shown to present low in vitro potency by exhibiting weak inhibition of PP1 and PP2A and therefore have been considered the initial biosynthetic products or self-protective precursors and the storage forms of DSTs [119]. Although a self-protecting role was hypothesized by Hu et al. (2017) [119], these toxin precursors have not been found in all OA-producing dinoflagellates. Anyway, on the occasion of cell death or cell disruption after ingestion by shellfish species, these compounds are readily hydrolyzed to form diol esters and the free OA or its isomers DTX-1 and DTX-2.

In addition to the hydrolysis of the sulfated diesters derivatives, which is a process that occurs throughout the shellfish digestive system before absorption, a second transformation process occurs in the digestive gland where toxins are accumulated. Most shellfish species rapidly transform OA and DTX-1 -,2 into FA esters derivatives [98]. The hydroxyl group in C7 of OA or DTX-1, -2 is esterified with FA of varying chain lengths and different levels of unsaturation [91,121]. Collectively known as DTX-3, the 7-O-acyl fatty acid derivatives were first identified in scallops from Japan in 1985 [91], and although the acylation reaction of OA and DTX-1 has been proved by in vivo and in vitro studies [95,122], the toxins esterification process is still unclear.

The most remarkable steps to understand shellfish esterification of OA and DTX-1, -2 have been made by Rossignoli et al. (2011) [98]. In their work, mussels (*M. galloprovincialis*) fed microcapsules containing OA showed that transformation occurs in the digestive gland cells, in the microsomal and mitochondrial subcellular fractions, by the action of acyl transferases enzymes. Afterwards, Konoki et al. 2013 [122] demonstrated a notable increase in the transformation of OA/DTX-1, -2 into DTX-3 in the presence of acyl-CoA, highlighting the need to further investigate and purify the OA-transforming enzymes. Indeed, this constitutes a major bottleneck in the understanding of DSTs biotransformation in shellfish. However, OMICs and particularly proteomics and genomics/transcriptomics disciplines, could have a major role in the identification of these enzymes. Genomic information (full sequenced genomes) is now available from a few commercial shellfish species (e.g., *M. galloprovincialis* and *M. gigas*), making possible performing wide BLAST sequence-alignment and phylogeny studies to discover potential acyl-transferase genes in shellfish. Similar strategy was used to screen and compare acyl-transferase gene families across vertebrates [123]. Moreover, the discovery of gene/protein sequence information of these enzymes opens several opportunities for computational investigations addressing substrate specificities of these toxin transforming enzymes and regulatory mechanisms. In the field of proteomics, most interesting results will likely arise from investigations targeting sub-proteome fractions enriched in OA and DTX-transforming enzymes.

Shellfish esterification of OA and DTX-1, -2 varies among species [100]. It is true that most species easily and rapidly conjugate the toxins free forms found in the dinoflagellate with shellfish FA, but certain species such as mussels (e.g., *M. edulis* and *M. galloprovincialis*), donax clam (*Donax* spp.) and queen scallops (*Aequipecten opercularis*) have been found to maintain relevant fractions of the toxins in the parental conformation [67,124,125,126]. Shellfish composition of FA, including saturated, SFAs, monounsaturated, MUFAs, and polyunsaturated, PUFAs, and its variability according to season, growth and other biological parameters is well known and has been characterized for a high number of bivalve mollusk populations [127,128]. Palmitic acid (16:0) followed by stearic acid (18:0) and myristic acid (14:0) are commonly the predominant SFA. Palmitoleic acid (16:1 ω7) and eicosenoic acid (20:1 ω7) are the most important MUFA, and regarding PUFA, the eicosapentanoic acid (EPA) (20:5 ω3) and docohexaenoic acid (DHA) (22:6 ω3) are particularly abundant. It is thus intuitive that conjugation of dinoflagellate toxins with shellfish FA leads to the formation of a wide range of toxin derivatives. Gerssen et al. (2011) [129] developed a library of OA derivatives and the necessary data to determine their presence in shellfish matrices by liquid chromatography coupled to mass spectrometry detection. Gerssen et al. (2011) [129] indicated 30 derivatives, resulting from the conjugation of OA and DTX-2 with shellfish FA. However, several other compounds may be formed either by the conjugation with DTX-1 or with other FA [130].

Few attempts have been made to correlate OA toxicity in shellfish tissues and FA content, with some positive relationships being observed with SFA [131]. But several studies are consensually indicating that esterification of toxins facilitates their elimination and decrease of shellfish toxicity [124,132]. Differences on elimination rates have been observed between shellfish species and between individual esters. For example, esters of DTX-1 can be more significantly slowly eliminated than esters of OA and DTX-2 [124], but it is the ability to transform or the lack of this ability to transform the parental toxins into acyl derivatives that causes the great implications to shellfish toxicity. Species like mussels, donax clams and queen scallops that tend to accumulate and maintain high toxins levels in the parental conformation remain toxic for long periods of time causing severe impacts to shellfisheries industry [133]. This is a research area in which we expect considerable advances in the near future, given that much technological barriers in metabolite analysis have been overcome, with new mass-spectrometry instruments enabling high accuracy and high-throughput analysis of complex biological matrices and advances verified also in computational and statistical methods to address data sparsity in high-throughput metabolomics approaches [134]. In summary, the analysis of the acyl derivatives of OA and DTX-1, -2 is of utmost relevance, as this information is needed for assessment of shellfish contamination. Moreover, it will be important to understand lipid metabolism in shellfish and its relation with toxin transformation mechanisms. Therefore, we anticipate in the future developments in the field of metabolomics and the development of high throughput methods specific for profiling DSTs and corresponding FA derivatives. Furthermore, integration of metabolomics with genomics and proteomics (and molecular transport processes) could lead to more insights with respect to the role of biotransformation and esterification in the process of inactivation and elimination of DSTs in shellfish.

## 7. Biomarkers of Exposure to DSTs

Biomarkers were defined by Amacher (2010) [135] as biometric measurements that provide critical quantitative information about the biological condition of an animal or individual being tested. These measurements are particularly useful to diagnose, predict, infer the cause, monitor the progression or recession of a disease/illness, or identify the outcomes of different types of stressors. Biomarkers could be divided into cellular, when referring to non-molecular characteristics of cells and tissues such as morphology, cellular integrity, cell/tissue damage and phagocytic activity, or molecular, when referring to protein and gene expression, protein activity and modification, sub-cellular localization of proteins and mRNA [136,137]. Moreover, biomarkers can also be classified as of exposure, effect and susceptibility [138]. Biomarkers of exposure are related with the characteristic of the contaminant or drug, whereas biomarkers of effect give information of the magnitude of the changes, and biomarkers of susceptibility can be used to describe how sensitive or vulnerable is an organism to a particular stress factor [138].

Concerning environmental monitoring and assessment of trophic contamination, sentinel organisms such as shellfish are often selected for such studies. The unique sessile lifestyle and filter feeding behavior of shellfish has been particularly important to carry temporal and spatial studies on the levels of contamination in coastal ecosystems [139]. Shellfish are also suitable for monitoring the occurrence of DSTs, since these animals feed on DST-producing microalgae and accumulate high levels of DSTs, as discussed in the previous sections. Consequently, shellfish are among the first animals to respond to the presence of these toxins in the environment and to exhibit cellular and metabolic changes [72,138]. DNA breaks, chromosome alterations or cell viability rates constitute potential genotoxic and cytotoxic markers of DSTs exposure in shellfish [138]. Moreover, transcriptomics and proteomics studies, reviewed in the previous section, have revealed a considerable number of potential molecular markers. Many genes/proteins were differentially expressed in the presence of DST-producing microalga and/or OA toxin, revealing a link with the toxic activity of DSTs. We highlight as candidate markers the genes/proteins from shellfish antioxidant system, since oxidative stress was reported as one of the main toxic outcomes of DSTs [138]. Among the genes/proteins of the antioxidant system that showed to be sensitive to DSTs are GPx and Se-GPx, CAT, SOD and GST [72]. The biological functions of these genes/proteins are reported in Table 1.

Another group of differentially expressed genes/proteins revealed by OMICs have functions in the metabolism (biotransformation) and elimination of these toxins. Several of these genes/proteins belong to the families of CYP450 and ABC transporters (Table 1). Moreover, these genes and proteins demonstrated to respond to the presence of DST-producing *P. lima* and OA therefore constitute important molecular markers of exposure to this toxin. For instance, *CYP2D14-like* was found up-regulated in *P. viridis* digestive gland at 2 and 6 h and significantly down-regulated in the gills after 6 h of exposure [73]. On the other hand, *CYP3A4* expression increased at 2 h in the gills but also at 12 h in both gills and digestive glands, while *CYP3L3* and *CYP2C8* were down-regulated at 12 h and upregulated at 6 h, respectively [73]. In scallop *A. irradians*, the exposure to OA induced the expression of genes encoding several ABC transporters (*ABCB10*, *ABCC5* and *ABCC1*) [140] and in contaminated *P. viridis P-GP* gene was also up-regulated [75]. *GST* and *ABC* genes can be pointed out as markers of short-term exposure, because their expression was modulated early to DSTs exposure. In turn, other genes reflect the effects of prolonged exposure to DSTs. Among those are genes involved in the NRF2 signaling pathway and inhibitors of apoptosis protein (*IAP*) (Table 1), which are activated in a later phase of exposure [84].

Similarly, genes from the immune system of shellfish could also be studied with regard to DSTs exposure (Table 1). Indeed, genes encoding C-type lectins (CLT-6), HSP90, metallothionein (MT), classical complement pathway C1q (mgC1q83 and mgC1q29), macrophage migration inhibitory factor (MIF) or fibrinogen-related protein (FREP) have been found to be modulated by DST-producing *P. lima* [82,84,141].

In summary, OMICs disciplines have been contributing to the discovery of relevant molecular makers of shellfish exposure and contamination with DSTs. These could be used to assist the current environmental monitoring programs and include biomonitoring to the analysis of toxic phytoplankton and the chemical analysis of DSTs. Molecular markers can have an important role in predicting, diagnosing and assessing the impact of DSTs in aquaculture species and particularly in shellfish species that are most affected by DSTs contamination. Still, biomarker research is in an initial stage of development, and we recognize that much more investigation must be developed before routine biomonitoring programs can be established in field.

Furthermore, we note that the advances in OMICs, in particular the establishment of high-throughput transcriptomics and proteomics, is progressively moving biomarker research from the earlier concept of disease-specific markers. These rely on the analysis of a few genes and proteins to characterize a particular health state, to the concept of disease-specific patterns [142], in which complex gene and protein profiling are instead used to characterize health states. These new approaches based on gene and protein profiling are expected to have more potential for the differentiation of health conditions and thus to differentiate the effects of distinct xenobiotic substances including drugs and toxins. Likely in the future, specialized libraries or repositories of high-throughput gene and protein expression data will be developed to assist the biological interpretation of new experimental data and in the identification of the main causes or stressors responsible of adverse health effects.

## 8. Concluding Remarks

DSTs may affect human health due to consumption of contaminated shellfish. To minimize the risk of acute intoxications, temporary bans of shellfish harvesting and/or farming have to be enforced whenever toxins exceed the safety limits. However, this reactive approach, which is in place in most coastal countries with the aim of protecting public health, has a severe impact on the shellfish industry. Furthermore, many countries in Africa, Asia or South and Central America lack the ability to enforce such banning policies. Therefore, research on novel and proactive strategies is of paramount importance. The involvement of OMICs in aquatic sciences and in research of marine biotoxins is seen with great expectation as relevant new data can be generated and elucidation of accumulation/elimination mechanisms achieved. From the most recent and most relevant results from OMICs in these areas, as discussed above, it is expected that OMICs may decisively contribute to:

- Further understanding of the molecular mechanisms associated with the toxicity of DSTs;
- Knowledge of the health effects associated to the chronic exposure of seafood consumers;
- Elucidating the mechanisms of transformation of DSTs in seafood, particularly in shellfish. There is an evident absence of molecular data concerning the bioactivity of the different DTX toxins and OA derivatives (esterified forms) relevant to the assessment of DSTs toxicity. There are other specific questions concerning the metabolic transformation of DSTs that can be elucidated employing metabolomics methods (e.g., chemical profile of FA-derivatives of OA and DTXs);
- OMICs will have a key role on the understanding the molecular processes that characterize bioavailability and bioaccessibility of DSTs, which is critical to promote an accurate risk assessment;
- Finally, proteomics can be particularly important in the investigation on the expression and accumulation of allergenic proteins in contaminated seafood and likely in assessing associations of DSTs with the protein matrix in contaminated seafood;
- Notwithstanding, OMICs breakthroughs in DSTs knowledge will be dependent on the improvement of the capacity of analysis of OMICs technologies. For instance, the access to thorough and well annotated genomic databases, particularly from marine invertebrates, will facilitate extracting biological information from complex gene and protein datasets. In proteomics, specific strategies, namely concerning protein fractionation, enrichment and isolation, are needed for capturing specific information about the metabolic pathways involved in DSTs toxicity and biotransformation.

## Figures and Tables

**Table 1:** List of candidate gene/protein markers of DST exposure in shellfish revealed by OMICs approaches.

Functional Categories	Molecular Marker	Biological Function	References
**Antioxidant system**	Superoxide dismutase (SOD)	removal of superoxide radicals	[72]
	Catalase (CAT)	hydrogen peroxide catabolic process	[72]
	Glutathione peroxidase (GPx, Se-GPx)	hydrogen peroxide catabolic process	[72]
	Glutathione S-Transferase (GST, GST-pi, GST-σ3, GST-ω)	glutathione peroxidase activity, xenobiotic catabolic process	[72,84,143]
**Metabolic detoxification**	Cytochrome P450 (CYP2D14, CYP3A4, CYP3L3, CYP2C8, CYP3A)	xenobiotic metabolic process, oxidoreductase activity	[73]
	ATP-binding cassette sub-family B member 10 (ABCB10)	transmembrane transport	[84]
	Multidrug resistance-associated protein (ABCC1, ABCG)	xenobiotic transmembrane transporter activity	[84,143]
**Transcription**	Cyclic AMP-dependent transcription factor (ATF4-like)	regulation of transcription by RNA polymerase II	[84]
	CREB/ATF bZIP transcription factor (CREBZF)	transcription regulation	[84]
	Nuclear factor erythroid 2-related factor (NRF2)	transcription factor that plays a key role in the response to oxidative stress	[84,143]
**Protein metabolism**	Inhibitor of apoptosis protein (IAP)	ubiquitin-dependent protein catabolic process	[84]
	Kelch-like ECH-associated protein 1 (KEAP1)	protein ubiquitination, cellular response to oxidative stress	[84,143]
**Cell structure**	Tubulin alpha-1C chain (TUBA1C)	microtubule constituent	[84,143]
	Tubulin beta-1 chain (TUBB1)	microtubule constituent	[84,143]
	Actin related protein (ARP 2, ARP 3)	mediates actin polymerization	[76]
**Immune response**	annexin-like (ANXA)	regulator of the inflammatory process	[76]
	Complement component 1q (mgC1q83 and mgC1q29)	pathogen recognition	[84]
**Regulation of metabolism and signal transduction**	Heat shock protein HSP 90 (HSP90)	regulation of specific target proteins involved for instance in cell cycle control and signal transduction	[84]
